# Editorial

**Published:** 1856-04

**Authors:** 


### EDITORIAL.

How ceaselessly play the long, invisible *Antennæ* of an Editor, day and night, summer and winter, in the street and on the highway, at opera, church or ball room, on the rail, in the steamship, carriage, car or omnibus, appropriating towards his capacious maw all that comes within the scope of their far outreachings. Omnivorous are they, taking their supplies from air, earth and ocean, filling up their wide Alembic, hour after hour, and turning out therefrom somewhat for the Pet which is the God of their idolatry, whether it be an eight by ten daily or a mammoth weekly—whether it boasts of a circulation of one or one hundred thousand, each one's paper is his world, occupying all his thoughts, and filling up even his dreams. Not a falsehood, or fact, nor custom nor accident, not a sermon nor a soiree, nor fire, nor murder, nor shipwreck, no cause of gladness or of gloom, no war of weapons or of words, no rumor of disaster or of death, nothing that is now or ever shall be, not an individual thing in the heavens above, in the earth beneath, or in the waters under the earth, but he tortures to make capital out of, for the uses of his all-shadowing journal.

But to come to a personal matter of fact, so as to leave no doubt of our meaning, and to show too, how keen the look-out and how stretching are the instincts of an Editor, what wide circuits he sometimes takes to enable him to bend occurrences to his uses, we may mention our own experience, one day lately in seeing in a familiar operation in Wall Street, one of the most rational cures for consumption ever proposed. The full rationale of the cure it may not be well to attempt describing at this time, but until we are moved to do so, our readers may amuse themselves by that most valuable of all methods of learning, self-thinking, thinking out things for one's self, what connection there is between any one familiar operation of Wall Street, and its adaptedness to the cure of consumptive disease, certain it is, the thought never occurred to us until within a year, certain is it, that it involves a principle, without whose application, no case of tubercular consumption ever was cured ; and we venture to say ever will be; and strange to tell, not one of the men who have been really successful in the treatment of this disease in either hemisphere, but owes his success to the application of this principle, and in proportion, too, to the extent to which it has been made.

